# Application of enzymes in the preparation of wheat germ polypeptides and their biological activities

**DOI:** 10.3389/fnut.2022.943950

**Published:** 2022-07-18

**Authors:** Ke Du, Shuangqi Tian, Hu Chen, Sensen Gao, Xianyou Dong, Feng Yan

**Affiliations:** ^1^College of Food Science and Technology, Henan University of Technology, Zhengzhou, China; ^2^Kemen Noodle Manufacturing Co., Ltd., Changsha, China; ^3^Angel Yeast Co., Ltd., Yichang, China

**Keywords:** wheat germ protein, enzymatic hydrolysis, peptides, biological activity, proteases

## Abstract

Wheat germ, a byproduct of wheat industrial processing, contains 30% protein and is a comprehensive source of plant-based protein. But a large amount of wheat germs are disposed of as waste every year. Wheat germ protein can be hydrolyzed into polypeptides with antioxidant, antihypertensive, anti-tumor, bacteriostatic and other activities. At present, researches on the hydrolysis of wheat germ protein and the preparation of bioactive peptides from wheat germ protein have attracted increasing attentions. However, the traditional protein hydrolysis method, protease hydrolysis, can no longer meet the market's needs for efficient production. Various auxiliary means, such as ultrasound, microwave and membrane separation, were applied to boost the yield and biological activity of wheat germ peptides by enzymatic hydrolysis. Under ultrasound and microwave, the protein structure may expand to increase the binding sites between enzyme and substrate and promote hydrolysis efficiency. Membrane separation is applied to separate products from enzymatic hydrolysate to reduce the inhibitory effect of the product on the hydrolysis reaction. The paper reviewed the hydrolysis methods of wheat germ protein and summarized the biological activity of wheat germ peptides to provide references for further study of wheat germ peptides.

## Introduction

Wheat (*Triticum aestivum* L.) is a kind of important grain cereals, and wheat seeds consist of endosperm, bran and germ. Wheat germ, accounting for 2%-3% of the total grain weight, is the part that breeds new life, and is rich in protein, fat, unsaturated fatty acids, dietary fiber, minerals, and vitamins (1). Wheat germ and bran are the most important by-products of the dry milling industry, and these by-products are not well utilized. At present, wheat germ is mainly used to produce wheat germ oil, and wheat germ protein is the most important by-product in the production of wheat germ oil. Defatted wheat germ contained 30% protein, which meet the requirements of FAO/WHO for high-quality protein (2–4). Wheat germ protein contained 18.9% globulin, 0.30%-0.37% glutenin, 30.0% soluble albumin, 14.0% malt soluble protein, and various essential amino acids such as lysine, threonine and methionine (5–7). It is one of the most abundant sources of plant protein (8), and it is easier to obtain higher nutritional value than animal protein sources (9, 10). Wheat germ protein might affect immune regulation, aging and inhibit tumors. It could be directly used to treat cholesterol reduction (11–13), but its allergens also need to be alerted (14).

Wheat germ proteins have many amino acid sequences with biological action and can be used to prepared bioactive peptides. The production of wheat germ peptides not only can increase the source of bioactive peptides, but also improve the utilization rate of wheat germ (15). Wheat germ powder was defatted and hydrolyzed to obtain wheat germ protein hydrolysates (WGPHs) (16). WGPHs contained free amino acids and polypeptides with different molecular weights, which could be purified by ultrafiltration, anion exchange chromatography, gel filtration chromatography, reversed-phase high performance liquid chromatography and other methods (16). According to molecular weight, peptides could be divided into 11,563–1512 Da (17.78%), 1512–842 Da (17.50%), 842–372 Da (27.38%) and 372–76 Da (30.65%) (17). The traditional enzymatic hydrolysis method was protease hydrolysis, such as alcalase alkaline protease, flavourzyme, papain, neutrase and protamex (18). However, the traditional method has the disadvantage of low hydrolysis efficiency. To overcome this shortcoming, several technologies, including ultrasonic (19), microwave (20) and membrane separation technology (21), were adopted to maximize the hydrolysis rate of protein and the utilization rate of enzyme.

More and more researchers have begun to study bioactive peptides to replace drugs to treat cancer (22), hypertension and other diseases. Bioactive peptides could be extracted from corn protein, soybean powder, tea seed powder, and so on (23–26). Wheat germ could release bioactive peptides under the action of protease, such as an anti-fatigue peptide, antihypertensive peptide, antioxidant peptide and antibacterial peptide (2). Wheat germ polypeptide had immune activity in treating celiac disease (27), and could be used as a natural antioxidant to destroy reactive oxygen species (28). Antioxidant peptides in wheat germ could inhibit ultraviolet-induced oxidative damage by promoting the activity of superoxide dismutase, glutathione peroxidase, catalase and ascorbate peroxidase (29). Electron beam irradiation (EBI) could destroy the secondary structure of protein, reduce the surface hydrophobicity of protein, and at the same time improve the antioxidant capacity of WGPHs (30, 31). However, heat and malondialdehyde treatment could reduce the free radical scavenging ability of wheat germ polypeptides (32). Therefore, the focuses of this paper were as follows: (1) The principles, advantages and disadvantages of preparation methods of wheat germ polypeptide; (2) The biological activity of wheat germ polypeptide and its current application direction.

### The preparation methods of wheat germ polypeptides

#### The preparation of wheat germ protein and selection of proteases

There are many impurities in hydrolysate (such as carbohydrate, pigment, etc), which are difficult to remove when the defatted wheat germ used as substrate (33). Therefore, the extraction of defatted wheat germ protein is more beneficial to the preparation of high purity polypeptide products. The preparation of wheat germ protein usually comprised the following steps (18): heating wheat germ 105 °C for 15 min to inactivate the endogenous enzymes of wheat germ, defatting with supercritical carbon dioxide to obtain defatted wheat germ (DWG) powder, extracting defatted wheat germ protein (DWGP) with alkaline solution (pH 9.5), adjusting pH to the isoelectric point of the wheat germ protein to precipitate the protein (34), and freeze drying to obtain the wheat germ protein powder. DWGP could also be extracted directly by reverse micellar extraction (35).

The peptide bond of protein was broken under the action of protease, and peptides with different molecular weight are generated, which were more easily absorbed by the human body. The degree of hydrolysis (DH) was used to indicate the degree of protein hydrolysis. If the DH value was higher, it means that the more peptide bonds were fractured and the proteins were hydrolyzed more thoroughly. With the extension of hydrolysis time, the degree of hydrolysis of protein gradually increased, while the molecular weight of peptide gradually decreased. However, peptides had the optimal molecular size or chain length (mainly dipeptide and tripeptide) and better functional characteristics (36–38). Therefore, it was necessary to find the suitable conditions for enzymatic hydrolysis. Proteases commonly used in enzymatic hydrolysis of wheat germ protein include flavor protease (18), Alcalase alkaline protease (19), papain (39), and so on. The enzymatic hydrolysis conditions of protease were different, and the properties of hydrolysate were different, as shown in Table 1.

**Table 1:** The enzymolysis of wheat germ protein and biological activities of peptides.

The substrate	Protease	The enzymolysis condition	Biological activities	Reference
DWGP	Alcalase 2.4 L FG	Protein isolate concentration 10%; enzyme–substrate ratio (E/S) = 0.4 AU/g; temperature 50 °C; time 6.0 h; pH 8.0	The EC_50_ values of DPPH, superoxide, and hydroxyl radicals were 1.30, 0.40 and 0.12 mg/mL; WGPH exhibited notable reducing power and strong chelating effect on Fe^2+^	Zhu et al. (7)
WGPHs	Proleatber FG-F	Protein isolate concentration 5%; temperature 55 °C; time 3.0 h; pH 9.5	The scavenging rate of DPPH radical was 81.11%	Cheng et al. (17)
DWG	Flavourzyme (500 LAPU*/g)	Enzyme to substrate ratio of 1.3% (w/w); temperature 50 °C; time 9 h; pH 7	Treatments solubilized 80% of the total proteins	(Claver and Zhou (18)
DWGP	Alcalase (1.325 × 10^5^ U/g)	Substrate concentration of 24.0 g/L; ultrasonic power of 24 W; sweep frequency of 24 ± 2 kHz; temperature 50 °C; time 120 min; pH 8.0;	Conversion rate of protein was 60.7%; and ACE inhibitory activity of peptide was 65.9%	Qu et al. (19)
DWGP	Alcalase 2.4 L FG (2.670 U/g)	Substrate concentration of 10.65 g/L; CEH-MS permeation flux of 0.011 L/min; Alcalase quantity of 0.51 g, effective volume of 0.4 L; temperature 50 °C; time 300 min; pH 9.0	Conversion rate of protein (65.21%); yield of peptides (34.10 g/g); the IC_50_ value of ACE inhibitory activity of peptides was 0.452 g/L	Qu et al. (21)
DWG	*Bacillus licheniformis* alkaline protease	Enzyme to substrate ratio of 0.5 wt.% temperature 50 °C; time 8 h; pH 9.0	The IC_50_ value of ACE inhibitory activity of peptides was 0.48 μg/mL	Matsui et al. (33)
Wheat germ albumin (WGA)	Papain (9.32 × 10^3^U/g)	Microwave power 600 W; temperature 65 °C; time 9 min; pH 7.0	In the range of pH 5.0-10.0, the nitrogen solubility of polypeptides was more than 96%	Tian et al. (39)
DWGP	Alcalase (2.4 AU/g)	Protein isolate concentration 5% (w/v); enzyme to substrate ratio of 1% (w/w); temperature 55 °C; time 200 min; pH 8.3	DH of 15.61 ±0.09%; metal chelating ability of 69.62 ± 0.96%	Zhu et al. (40)
DWGP (pretreated by ultrasound at 1500 W for 20 min)	Alcalase (132,507 U/g)	Substrate concentration of 1%(w/v); enzyme to substrate ratio of 3500 U/g; temperature 50 °C; time 90 min; pH 8.0	ACE inhibitory activity of DWGP hydrolysate was increased by 21.0-40.7%	Jia et al. (41)
DWGP (pretreated by ultrasound at 600 W for 10 min)	Alcalase	Substrate concentration of 1.17%; enzyme to substrate ratio of 2.18% (E/S); temperature 50 °C; time 88.14 min; pH 9.0	ACE inhibitory activity of the hydrolysate was 68.96%	Huang et al. (42)
Wheat germ protein isolates (WGPI)	Alcalase 2.4 L FG (2.670 AU/g)	Substrate concentration of 1.2% (w/v); enzymatic membrane reactor (EMR) permeation flux of 0.005 L/min and turn into 0.011 L/min after 10 min; alcalase quantity of 110 μL, effective volume of 0.4 L; temperature 50 °C; time 300 min; pH 9.0	The conversion rate of protein (55.3%) increased by 36.17% and the IC_50_ value of ACE inhibitory activity of peptides (0.50 mg/mL) reduced by 30.6%	He et al. (43)
DWG	*Bacillus Subtilis* B1	Inoculum size 8%; fermentation temperature 31 °C; fermentation time 48 h	The yield of defatted wheat germ peptides was 8.69 mg/mL; The EC_50_ values of DPPH, hydroxyl and superoxide anion radicals were 3.16 mg/mL, 6.04 mg/mL and 7.46 mg/mL	Niu et al. (44)
Wheat gluten protein	Alcalase 2.4 L	Substrate concentration of 10% (w/v); enzyme to substrate ratio of 0.1 AU/g (E/S); temperature 50 °C; time 60 min; pH 8	WGPHs treatment was able to reduce cell proliferation and improve the cellular anti-inflammatory microenvironment; WGPHs increased GSH levels and reduced the NO production	Cruz-Chamorro et al. (45)
Wheat gluten	*Pseudomonas aeruginosa* (Papro A)	Substrate concentration of 5% (w/w); enzyme to substrate ratio of 1500 U/g; temperature 45 °C; time 6 h; pH 7.0	The IC_50_ values of SAGGYIW and APATPSFW were 0.002 mg/mL and 0.036 mg/mL	Zhang et al. (46)
DWGP	Pronase	Substrate concentration of 1% (w/v); enzyme to substrate ratio of 2% (w/w); temperature 50 °C; time 4 h; pH 7.5	The activity of inhibiting *H. pylori* adhesion to gastric epithelial cells was 51.7 ± 6.8% at 10 mg/mL; and the minimum anti–adhesive concentration was 0.31 mg/mL	Tanikawa et al. (47)
Defatted wheat germ globulin (DWGG)	Alcalase (200 U/mg)	Substrate concentration of 4%; enzyme to substrate ratio of 10000 U/g (E/S); temperature 50 °C; time 3 h; pH 8.0	The hydrolysate had immunomodulatory activity with respect to lymphocyte proliferation, phagocytosis of neutral red and secretion of pro–inflammatory cytokines	Wu et al. (48)

#### Protease hydrolysis

##### Traditional protease hydrolysis

The traditional enzymatic hydrolysis method was through protease hydrolysis without any auxiliary methods. Zhu et al. (40) hydrolysated DWGP by flavourzyme, alcalase alkaline protease and papain. They found that alcalase alkaline protease had the highest DH value and metal chelation activity. After 180 min, the rate of enzymolysis slowed down, but the metal chelation activity of hydrolysate reached the maximum of 69.62±0.96% at 200 min, and then decreased with the progress of hydrolysis reaction (40). Wang et al. (16) found that the maximum calcium binding capacity of WGPHs was 48.26 ± 0.53% after hydrolyzing DWGP for 240 min. When the hydrolysis time exceeded 240 min, the calcium binding capacity decreased rapidly. The amino acid sequence FVDVT (PHE-ASP-Val-THR) of WGPHs was obtained after purification, and its calcium binding capacity reached 89.94 ± 0.75%, which was 86.37% higher than that of WGPHs (16). Protease hydrolysis had many advantages, such as high biosafety, no toxic and harmful by-products, controllable enzymatic hydrolysis conditions, economy and high efficiency. However, the conventional enzymatic hydrolysis method takes too long, and as time goes on, the activity of enzyme will decrease. It is necessary to look for auxiliary methods to solve these problems.

##### Ultrasonic assisted enzymatic hydrolysis

There were two main methods of ultrasound-assisted enzymatic hydrolysis: sonication during enzymatic hydrolysis and ultrasound pretreatment of DWGP and preparation of enzymatic hydrolysates. At present, most of the research is about ultrasonic pretreatment of wheat germ protein. The effects of ultrasonic on protein enzymatic hydrolysis mainly included the change of protein structure, enzyme activity and enzymatic hydrolysis process. The porous protein structure was obtained by ultrasonic treatment to break the hydrogen bonds and other secondary bonds between protein molecules (49). After ultrasonic treatment, the β-turns and β-sheets of DWGP transformed, and the hydrophobic groups of proteins were exposed, which made them more susceptible to alkaline protease, while the contents of hydrophobic amino acids related to angiotensin converting enzyme (ACE) inhibitory activity increased (41, 50, 51). Liu et al. (51) found that ACE inhibitory activity of protein enzymatic hydrolysate was increased by 23.96% after 10 min of ultrasonic pretreatment with 600 W. Huang et al. (42) confirmed that after DWGP was pretreated with 600 W ultrasonic for 10 min, the ACE inhibitory activity could increase by 32.14% compared with the control group. Ultrasonic wave could change the enzyme activity by changing the structure of the enzyme. When the ultrasonic wave was strong, the enzyme would be destroyed and its activity would decrease or disappear (52). Ultrasonic technology could be divided into probe ultrasonic instrument and ultrasonic cleaning instrument. Both ultrasonic and temperature could affect the enzymatic hydrolysis effect, especially at low temperature. DWGP solution was pretreated by ultrasonic cleaning instrument at 20 °C for 30 min at 1200 W power, and pretreated by ultrasonic instrument with 600 W probe at 20 °C for 10 min. Under the same conditions, the concentration of peptide obtained by enzymatic hydrolysis was 231.019 μg/mL or 231.320 μg/mL (53). Ultrasonic wave could generate mechanical heat and accelerate the hydrolysis reaction of enzyme and substrate, which not only shortened the reaction time, but also improved the utilization rate of enzyme and the conversion rate of the substrate (19). However, in the specific experimental process, attention should be paid to the temperature change during ultrasonic treatment, because long-term ultrasonic treatment will cause the temperature to rise sharply, adversely affecting the heat-sensitive materials. In addition, the application of ultrasonic wave in enzymatic hydrolysis process of DWGP should be lucubrated.

##### Microwave-assisted enzymatic hydrolysis

The microwave radiation region are between infrared radiation and radio wave, and the frequencies of microwaves is between 0.3–300 GHz. The matrix must contain dipole or ionic material to react and generate energy (54). Microwave technology was mainly used for stabilization of wheat germ, because microwave treatment can inactivate lipase of wheat germ, reduce water content and prolong the storage period of wheat germ. However, too high microwave power might have adverse effects (20, 55). The heating of proteins by microwave energy was achieved by the rotational absorption of microwave energy by bipolar water molecules and the translation of ionic components into proteins. Microwave could also change the flexibility of the enzyme, increase the activity of the protease, and accelerate the rate of enzyme hydrolysis (56). Microwave treatments improved the solubility of protein, the recovery rate of protein, degree of hydrolysis and the scavenging activity of 2,2'-azino-bis (3-ethylbenzthiazoline-6-sulphonic acid) (ABTs) free redical (57). Compared with untreated proteins, the hydrolysates treated by microwave showed higher ACE inhibitory activity and lower immunological activity, and the hydrolysate also contained a large amount of antioxidant peptides (58, 59). Tian et la. (39) hydrolyzed wheat germ protein under microwave power of 600 W and finally obtained the polypeptide with solubility >96%. In order to accelerate the enzymatic hydrolysis reaction under the microwave environment, the protease could be immobilized on the recyclable magnetite beads (immobilized enzyme). Magnetite beads absorbed microwave radiation, and the immobilized enzymes hydrolyzed protein faster than free enzymes, therefore the immobilized enzymes could be easily separated and reused (60). Microwave-assisted enzymatic hydrolysis can overcome the shortcoming of conventional enzymatic hydrolysis time-consuming and reduce the immune response of polypeptide. However, the application of microwave in wheat germ protein hydrolysis is not extensive, so it is necessary to study the changes of polypeptide of wheat germ protein after microwave treatment.

##### The coupling of enzymatic hydrolysis and membrane separation

**Figure 1:**
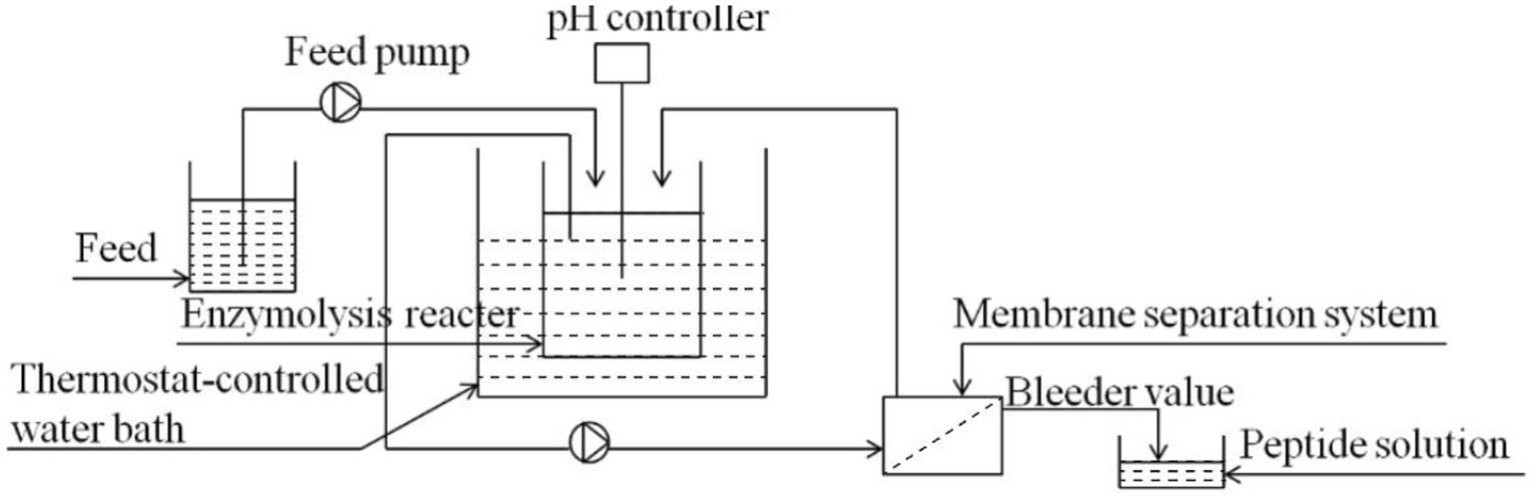
Continuous coupling of enzymatic hydrolysis and membrane separation (CEH-MS) set-up (21).

**Figure 2:**
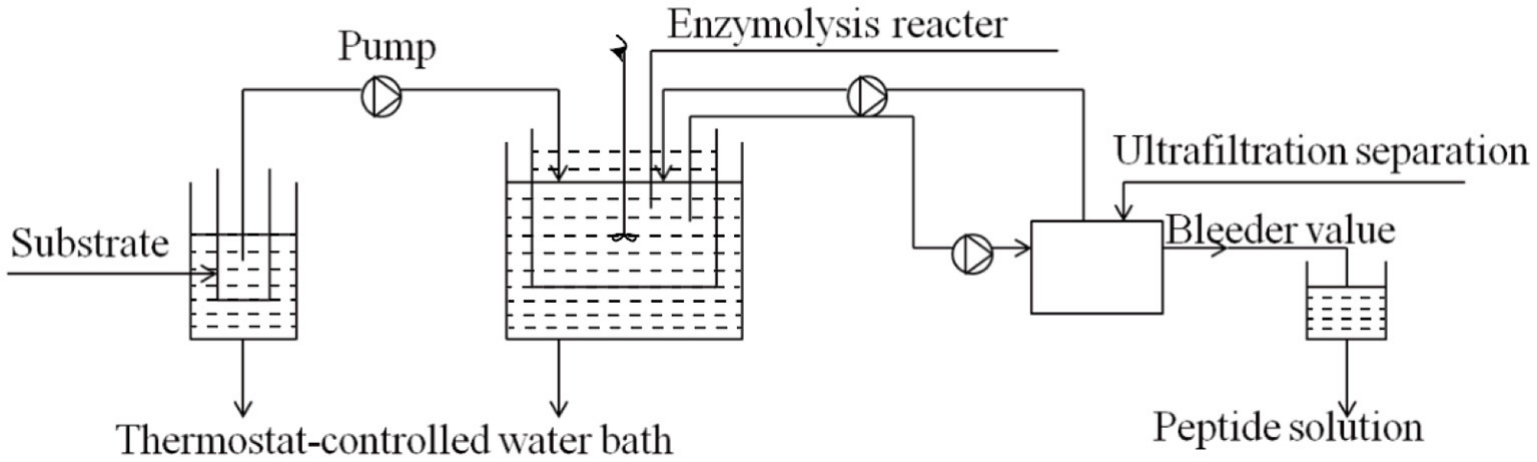
A schematic of enzymatic membrane reactor system for wheat germ protein isolates peptides preparation (44).

In the process of enzymatic hydrolysis, the accumulation of hydrolysis products inhibited the reaction rate. The reaction model was shown in Figure 1. The low concentration of substrate would make the model reach a stable state, while the high substrate concentration would lead to an increase in the number of peptide bonds. At that time, the system of continuous coupling of enzymatic hydrolysis and membrane separation (CEH-MS) was unstable, which aggravated the accumulation of unhydrolyzed protein (21). In order to ensure the smooth progress of enzymatic hydrolysis, it is necessary to strictly control the substrate concentration and clean the model on time. Membrane separation technology could be used to separate and purify peptides from enzymatic hydrolysate, as shown in Figure 2. The wheat germ protein solution was enzymatically hydrolyzed in a constant temperature-controlled reactor. The enzymatic hydrolysis products pass through the ultrafiltration membrane under the pressure of the pump, the target peptide was collected on the membrane module, and the macromolecules return to the reactor. The reactor combined protease solution and polypeptide extraction, and the reaction efficiency was significantly improved (43).

#### Fermentative method

**Figure 3:**
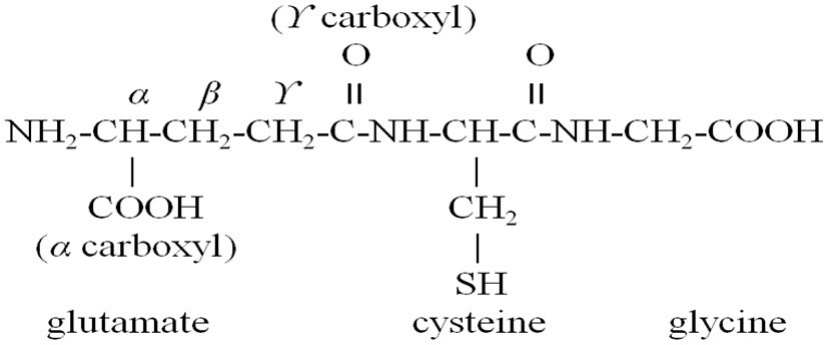
The formula of glutathione.

Wheat germ protein could be hydrolyzed by protease, and wheat germ polypeptide could also be directly prepared by microbial fermentation. *Bacillus subtilis* B1 hydrolyzed DWGP under the conditions of inoculation amount of 8%, fermentation temperature of 31 °C and fermentation time of 48 h. The yield of wheat germ peptide was 8.69 mg/mL, which showed high antioxidant activity (44). WGPHs achieved the purpose of antioxidation by scavenging free radicals, increasing glutathione level and inhibiting the production of nitric oxide (45). Glutathione consisted of glutamic acid, cysteine and glycine, which could be obtained by gene cloning (61), and its molecular formula was shown in Figure 3.

## The biological activity of wheat germ peptides

### Antioxidant activity

During the normal aerobic metabolism, organisms can produce free radicals, which can cause cell damage and even lead to atherosclerosis, arthritis, diabetes and cancer. Wheat germ polypeptide could be used as a natural antioxidant, because it had high free radical scavenging activity and strong lipid oxidation inhibition (62, 63). The ability of polypeptide to reduce and scavenging free radicals could be used to achieve the purpose of antioxidation (64). Zhang et al. (65) found that the wheat germ polypeptides had the activity of scavenging 1,1-Diphenyl-2-Picrylhydrazyl (DPPH) free radicals, and the peptides of WG-P-4 (Gly-Pro-Phe, GLy-Pro-Glu, and PHE-Gly-Glu were the major peptides of WG-P-4) with the highest antioxidant activity. The IC50 values for DPPH were found to be 1.17 mg/mL. Karami et al. (66) hydrolyzed wheat germ protein with pepsin and obtained the amino acid sequence of the antioxidant peptide with the highest clearance rate of 2,2'-azinobis-(3-ethyl-benzothiazoline-6-onate) (ABTs) as KELPPSDADW.

Yin et al. (67) tested non-steroidal anti-inflammatory drugs (NSAID_S_) on rats, and they found that the malondialdehyde (MDA) value of gastric mucosa, which caused damage and oxidative stress in small intestine of rats, was increased. Wheat peptides could enhance the activity of glutathione peroxidase (GSH) and reduce oxidative stress. Wheat peptides prevented gastric damage caused by NSAID_S_ by reducing oxidative stress and nitric oxide levels. Low concentration of wheat polypeptide solution had high activity and could protect gastric mucosa after oral administration (67, 68). At the same time, wheat peptides significantly decreased the activities of superoxide dismutase (SOD) and glutathione peroxidase (GSH) (69). In most organisms, superoxide anion radical can be easily converted to hydrogen peroxide by superoxide dismutase. In the absence of transition metal ions, hydrogen peroxide is quite stable, but it will produce hydroxyl radicals, which will attack and destroy every molecule in a living cell in the presence of metal ions such as iron or copper. WGPHs could scavenge superoxide anion, and the scavenging effect was enhanced with the increase of concentration (22). Ultraviolet radiation caused the generation of reactive oxygen species, such as superoxide anion radical, hydroxyl radical, and hydrogen peroxide. Scavenging reactive oxygen species could protect skin cells from the damage of ultraviolet radiation (70). In addition, reactive oxygen species was also the main cause of food spoilage (71). The antioxidant activity of wheat germ peptides could prevent their harm (72).

### Antihypertensive

Hypertension is an important risk factor of cardiovascular disease. Angiotensin converting enzyme (ACE) inhibitors, angiotensin II receptor blockers (ARBs), calcium channel blockers (CCBs), beta blockers and diuretics were all antihypertensive drugs (73). Angiotensin converting enzyme (ACE) was a dipeptide carboxypeptidase, which catalyzed the hydrolysis of angiotensin I to angiotensin II in the renin-angiotensin system. In the case of abnormal metabolism, ACE would catalyze degradation and inactivation of bradykinin, a vasodilator, which lead to trigger excessive levels of angiotensin II and lead to hypertension (46, 74). The antihypertensive activity of wheat germ was due to the ACE inhibitory peptide produced after hydrolysis of protein (75), and the dominant peptide of ACE inhibitory peptide was Ile-Val-Tyr (Ivy) (76). The activity of ACE obtained by different proteolytic methods was various (77, 78). The activity of ACE inhibitory peptide in wheat germ enzymatic hydrolysate obtained by Qu et al. (19) was 65.9%, while the activity of ACE inhibitory peptide in wheat germ enzymatic hydrolysate pretreated by Jia et al. (41) ultrasonic increased by 21.0-40.7%. Phenolic compounds and dipeptides with large side chains and hydrophobic residues could effectively inhibit ACE activity, and tripeptide with the same C-terminal as ACE inhibitory peptide could also inhibit ACE activity (79). ACE inhibitory peptide has remarkable effects in alternative drugs for treating hypertension, but it is usually necessary to take antihypertensive drugs for life, and further clinical trials are needed to determine whether ACE inhibitory peptide will increase the risk of cancer.

### Anticancer

In the past, the activity of glucose metabolism enzymes such as hexokinase, glucose-6-P dehydrogenase (G6PD) and transketoolase were controlled to inhibit the formation of cancer cells. The new effective natural and synthetic anticancer drugs mainly inhibited glucose-ribose conversion rather than the synthesis of nucleic acid purines and pyrimidines (80). Lunatic, which had the same sequence as soybean and barley, could be extracted from wheat. Lunasin was a unique 43-amino-acid cancer prevention peptide, which binded closely to deacetylated histone, destroying the kinetics of histone acetylation-deacetylation and leading to cell death, but this only occurred in transforming cells and does not affect normal cells. The polypeptide extracted from transgenic wheat had higher anticancer activity (81–83).

A fermented wheat germ extract (MSC) had immune activity on cancer cell system and could inhibit the synthesis and transfer of nucleic acid RNA of cancer cells (84). Methoxy-ρ-benzoquinone (MBQ) and 2,6-dimethoxy-ρ-benzoquinone (DMBQ) were two potential anticancer compounds. During the fermentation of wheat germ, the β-glycosidic bond of hydroquinone glycoside in wheat germ was cleaved by β-glucosidase of saccharomyces cerevisiae, and MBQ and DMBQ were produced (85). MSC could be used as a safe dietary supplement (86), and the addition of highly active β-glucosidase and peroxidase in fermentation broth could improve the yield of MBQ and DMBQ (87).

#### Others

Numerous proteins with anti-microbial activity can protect plants against pathogens, for example, lectins, pathogenesis-related proteins, hydroxyproline-rich glycoproteins, cyclophilin-like proteins, ribosome-inactivating proteins, and protease inhibitors (88, 89). On the other hand, wheat had anti-microbial peptides (AMPs) which showed higher resistance to microbial contamination (90). The main characteristic of AMPs was cationic, which enabled them to interact with microbial membranes directly or through a receptor-mediated mechanism, thus increasing the permeability of plasma membranes. Wheat peptides were resistant to most pathogens, such as brown rust, stem rust, and root rot (91, 92). In addition, it could also inhibit the adhesion of *helicobacter pylori* to gastric epithelial cells and prevent *helicobacter pylori* infection (93)).

Wheat peptide could protect rats from ethanol-induced gastric mucosal injury by inhibiting inflammation response, improving gastric mucosal blood flow, inducing endogenous molecules for cytoprotective, and maintaining the integrity of the gastric mucosal barrier (94). Wheat auxin-releasing peptides could effectively stimulate the secretion of auxin and improve gastric digestion (47). In addition, the combination of wheat peptides with fucoidan (WPF) would speed up the repair of gastric mucosa, which was due to WPF-nduced phosphorylation of a transmembrane receptor tyrosine kinase (EGFR) to protect gastric epithelial cells (95, 96). The process of preventing ethanol-induced gastric mucosal damage would not produce side effects (97). WPF could also improve the distribution of intestinal microorganisms and promote the production of short–chain fatty acids (SCFAs), thus alleviating chronic gastritis (98).

Arginine-valine-phenylalanine (RVF) derived from wheat germ peptide could prevent or delay the progression of neurodegenerative diseases (99). Wheat protein was rich in glutamine and proline residues, which could be separated and purified into opioid active peptides for treating neurological disorders (100). Wu et al. (101) found that glutelin (Gll) of wheat germ could enhance immune function by promoting lymphocyte proliferation. Wu et al. (48) hydrolyzed defatted wheat germ globulin (DWGG), and found that alcalase hydrolysate (AH) had the strongest immunomodulatory activity on lymphocyte proliferation, phagocytosis of neutral red and secretion of pro-inflammatory cytokines. The activities of polypeptide are shown in Table 1.

## Conclusion

So far, the reprocessing and utilization of industrial by-products is a way to reduce waste of resources and protect the environment. As a byproduct of dry milling industry, the nutritional value of wheat germ is often underestimated. Wheat germ protein is a kind of high quality protein. Wheat germ polypeptide has various biological activities, and is widely used in the fields of medicine, beauty and food. Bioactive peptides can be produced by enzymatic hydrolysis of wheat germ protein. The common proteases include alcalase alkaline protease, flavourzyme, papain, neutrase, and protamex. The enzymatic hydrolysis method using only protease is simple but inefficient. Microwave or ultrasound-assisted hydrolysis can improve hydrolysis efficiency, and different assisted methods enhance the bioactivity of peptides to various degrees. Although assisted enzymatic hydrolysis can shorten the time, microwave or ultrasound-assisted hydrolysis will produce noise, and need to find the best parameters when using these methods.

Wheat germ polypeptide has antioxidant, antihypertensive, anticancer activities, and can be used as natural medicine. Antioxidant peptide can also be used as a food additive. However, the research and mechanism of wheat germ polypeptide in drug production is still in the stage of animal experiment, and no side effects of wheat germ polypeptide have been found. Therefore, the researches on the extraction and activity of wheat germ polypeptide still need further study. Because the chain length of wheat germ polypeptide will affect its biological activity, it is necessary to separate and purify WGPHs to obtain the polypeptide with the highest activity. The cost of separation and purification is high, and bioactive peptide can not be fully absorbed and utilized. Then, the future research directions are as follows: (1) Researchers need to deeply understand whether wheat germ polypeptide has negative effects as a drug; (2) In the future, researchers will find a more effective and economical method for separation and purification; (3) Researchers need to find a new method to make more effective use of wheat germ polypeptide; (4) In-depth analysis of amino acid sequences of bioactive peptides.

## Author contributions

KD: conceptualization, software, and writing-original draft preparation. ST: funding acquisition, writing-reviewing and editing. HC: visualization and investigation. SG: supervision and project administration. XD: funding acquisition and supervision. FY: writing-reviewing, modify and editing. All authors contributed to the article and approved the submitted version.

## Conflict of interest

HC and SG were employed by Kemen Noodle Manufacturing Co., Ltd., and XD was employed by Angel Yeast Co., Ltd. The remaining authors declare that the research was conducted in the absence of any commercial or financial relationships that could be construed as a potential conflict of interest.

## Publisher's note

All claims expressed in this article are solely those of the authors and do not necessarily represent those of their affili-ated organizations, or those of the publisher, the editors and the reviewers. Any product that may be evaluated in this article, or claim that may be made by its manufacturer, is not guaranteed or endorsed by the publisher.
